# *Saccharomyces boulardii* CNCM I-745 synergizes with the small intestinal microbiota to boost AhR signaling in celiac disease

**DOI:** 10.1080/19490976.2026.2664640

**Published:** 2026-05-01

**Authors:** Kelly R. Kan, Marco Constante, Sara Rahmani, Gaston H. Rueda, Mark Wulczynski, Maria Ines Pinto-Sanchez, Xavier Roux, Premysl Bercik, Heather J. Galipeau, Alberto Caminero, Elena F. Verdu

**Affiliations:** a Department of Medicine, Farncombe Family Digestive Health Research Institute, McMaster University, Hamilton, Ontario, Canada; b Department of Biochemistry and Biomedical Sciences, Faculty of Health Sciences, McMaster University, Hamilton, Ontario, Canada; c School of Biomedical Engineering, McMaster University, Hamilton, Ontario, Canada; d Microbiology Department, Biocodex, 3 Chemin d'Armancourt, Compiègne, France

**Keywords:** Celiac disease, *Saccharomyces boulardii*, aryl hydrocarbon receptor (AhR), tryptophan metabolism, protein degradation, probiotics

## Abstract

Celiac disease (CeD) is an immune-mediated condition that leads to small intestinal villous atrophy and is driven by dietary gluten in individuals carrying HLA-DQ2 and DQ8. Microbial factors have been implicated in both the onset of CeD and persistent symptoms (non-responsive CeD) after the gluten-free diet (GFD), through mechanisms including impaired tryptophan metabolism and aryl hydrocarbon receptor (AhR) pathway activation. Although probiotics have been shown to be safe in CeD, there are currently no clinical recommendations for strains that target disease-related mechanisms. We here demonstrate that *S. boulardii* activated the AhR pathway in gluten-sensitized mice expressing HLA-DQ8, improving gluten-immunopathology. Mechanistically, *S. boulardii* enhanced the CeD patients' microbiota capacity for AhR activation when duodenal indole-producing commensals, such as *Lactobacillus reuteri*, were present. Our study provides preclinical evidence that *S. boulardii* CNCM I-745 targets a microbial deficiency previously described in CeD through modulation of microbial tryptophan metabolism. The findings encourage clinical testing of *S. boulardii* in CeD to prevent or better treat non-responsive cases.

## Graphical abstract

**Figure:**
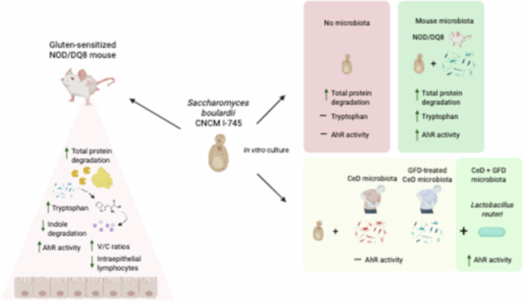

## Introduction

Celiac disease (CeD) is an immune-mediated food sensitivity, with autoimmune features, affecting the proximal small intestine and many extra-intestinal organs.^1^ The inflammatory CD4^+^ T cell response is driven by the ingestion of proteins from the prolamin family found in wheat, rye and barley, collectively called gluten, in genetically susceptible individuals who carry HLA-DQ2 or HLA-DQ8.^2^ While HLA allotypes and gluten ingestion are necessary for CeD onset, about 2%–3% of HLA-DQ2/DQ8 carriers will develop CeD,^3^ suggesting that other factors, including microbial, could play a role in disease pathogenesis.^4^^,^^5^

The gut microbiota metabolizes dietary tryptophan into ligands of the aryl hydrocarbon receptor (AhR),^6^ a ubiquitously expressed nuclear transcription factor which regulates the expression of target genes including *Cyp1a1, Il22,* and *Il17.*^7^ As such, the AhR pathway modulates intestinal immune and barrier functions and plays a large role in the balance between homeostasis and inflammation.^7^ In 8 patients with active CeD consuming gluten, we previously showed that microbial tryptophan metabolism and AhR pathway activation are impaired, and partially restored following treatment with a gluten-free diet (GFD).^8^ Combined with mechanistic preclinical studies, the results support the use of microbial therapies that target this pathway, to either accelerate mucosal recovery or improve inflammation in non-responders to the GFD. The latter constitute up to 30% of patients on the GFD,^9^^,^^10^ that either do not respond initially or reexperience symptoms, likely due to inadvertent contaminations.

Microbial therapeutics, including probiotics, may enhance both clinical and mucosal responses in patients with CeD.^11^^,^^12^ While probiotics have been used with some clinical success in improving gastrointestinal symptoms in CeD, no specific evidence-based recommendations can be given to date, because studies are small, highly heterogeneous and do not base the choice of the probiotic on the potential modulation of a disease-associated mechanism.^12^ This prompted us to investigate mechanistically a well-characterized probiotic, *Saccharomyces boulardii* CNCM I-745, which has been reported to improve intestinal barrier function,^13^ modulate intestinal microbiota composition^14^ and function,^15^ as well as improve infectious and acute diarrhea.^16^^,^^17^ Given that *S. boulardii* has been shown to boost overall protein degradation in the intestine,^18^ we studied its capacity to improve tryptophan metabolism, AhR pathway activation, and gluten-immunopathology in a mouse model expressing the disease susceptibility haplotype, HLA-DQ8. To explore mechanisms, the interactions between *S. boulardii* and small intestinal microbiota in relation to tryptophan metabolism were investigated *in vitro* using microbiota from both mice and CeD patients. Mice treated with *S. boulardii* had less severe gluten immunopathology, which was paralleled by increased AhR pathway activation. *S. boulardii* enhanced AhR activation by microbiota from both mice and CeD patients. However, for the latter, the effect required the presence of *Lactobacillus reuteri*, an AhR-agonist producing microbe. Our results suggest that probiotics such as *S. boulardii* CNCM I-745, targeting pathways dysregulated in CeD, could be considered for improving response to a GFD after clinical validation.

## Materials and methods

### Mouse model and overall design

Male and female specific pathogen free (SPF) non obese diabetic AB° DQ8 (NOD/DQ8) transgenic mice (8–11 week old) were maintained on a gluten-free diet for at least 2 generations (Envigo, TD.05620)^19^ (*n* = 6–8 per group as detailed below). Mice were sensitized with 500 μg of pepsin-trypsin digest of deamidated gliadin (PT-gliadin) and 25 μg of cholera toxin (Sigma-Aldrich, St. Louis, MO) by oral gavage once a week for 2 weeks, as previously described.^20^ One week following sensitization, mice were orally gavaged with 10 mg of gluten (Sigma-Aldrich) dissolved in acetic acid every 2 d for 3 weeks (gluten-sensitized). Non-sensitized mice (naïve) were used as controls.

### Probiotic treatment and pharmacological inhibitor of AhR

**Figure 1.**
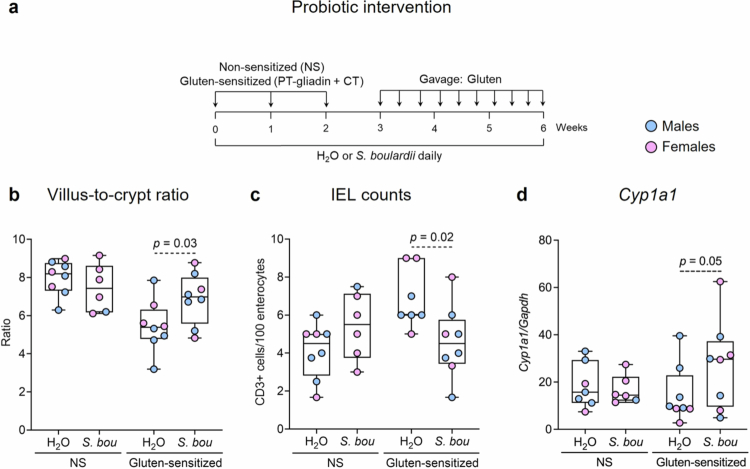
*S. boulardii* treatment reduces gluten immunopathology in NOD/DQ8 mice. (a) Experimental design for *S. boulardii* treatment of NOD/DQ8 mice. NOD/DQ8 mice were not sensitized (non-sensitized) or sensitized with PT-gliadin and cholera toxin once a week for 2 weeks. One week following sensitization mice were gavaged with gluten every 2 d for 3 weeks (gluten-sensitized). Non-sensitized or gluten-sensitized mice were gavaged with water (H_2_O) or *Saccharomyces boulardii* (*S. bou*) daily for 6 weeks. (b) Villus-to-crypt ratios, (c) intraepithelial lymphocyte counts, and (d) *Cyp1a1* expression in the small intestine of non-sensitized or gluten-sensitized mice treated with water or *S. bou*. Data are presented as median with interquartile range and whiskers extending from minimum to maximum, where each dot represents an individual mouse. Significant differences were evaluated using a estimated marginal means and custom contrasts using emmeans.

To determine the effect of the probiotic on gluten immunopathology, non-sensitized and gluten-sensitized mice were treated with 3 g/kg of *S. boulardii* CNCM I-745 (*S. bou*), as done previously,^21^ or water (H_2_O) daily for 6 weeks (gluten-sensitized + H_2_O *n* = 8, gluten-sensitized + *S. bou n* = 8, non-sensitized + H_2_O *n* = 8, non-sensitized + *S. bou n* = 6; Figure 1a and Supplementary Figure S1a).

**Figure 2.**
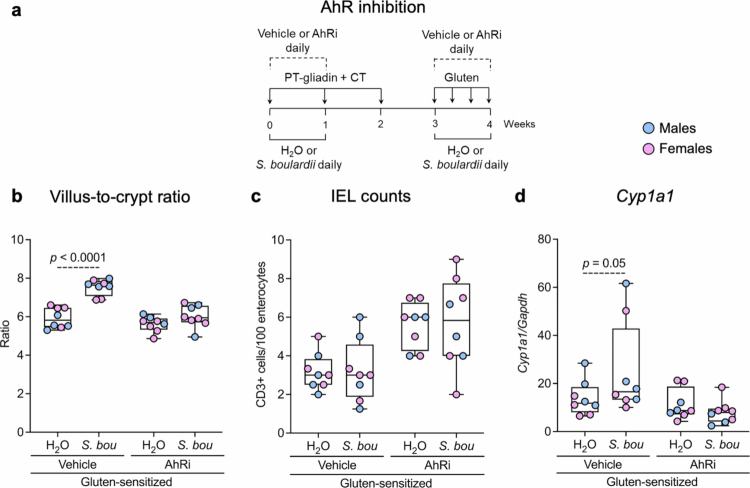
Beneficial effects of *S. boulardii* treatment are mediated through the AhR pathway in NOD/DQ8 mice. (a) Experimental design for AhR inhibition of NOD/DQ8 mice. Gluten-sensitized NOD/DQ8 mice were gavaged daily for 1 week of the sensitization phase and the challenge phase with *S. boulardii* (*S. bou*) or water (H_2_O), and with vehicle or CH-223191 (AhRi). (b) Villus-to-crypt ratios, (c) intraepithelial lymphocyte counts, and (d) *Cyp1a1* expression in the small intestine of mice treated with vehicle or AhRi receiving water or *S. bou*. Data are presented as median with interquartile range and whiskers extending from minimum to maximum, where each dot represents an individual mouse. Significant differences were evaluated using a estimated marginal means and custom contrasts using emmeans.

To investigate the potential role of the AhR pathway, additional gluten-sensitized mice were gavaged daily for the first week of the sensitization phase and the challenge phase with *S. boulardii* or water, and with vehicle (dimethyl sulfoxide, Sigma-Aldrich) or AhR inhibitor (CH-223191, Cedarlane) (*n* = 8 per group; Figure 2a and Supplementary Figure S3a). The duration of probiotic and pharmacological treatment was ethically required to be shorter given the known detrimental effect of inhibition of the AhR pathway.^22^ All experiments were conducted with approval from the McMaster University Animal Care Committee. After probiotic and pharmacological treatments, mice were sacrificed, and duodenal contents were collected by passing 250 μL of phosphate-buffered saline (PBS) through the duodenum. The duodenum was then opened longitudinally, and the epithelium and mucous layer were scraped and separated from the mucosa and resuspended in 250 μL of PBS. Proximal jejunum tissues were collected for preservation in RNALater and histological evaluation.

### Gene expression analysis in mouse small intestine

Total RNA extraction from proximal jejunum tissues was performed with the RNeasy Mini Kit (Qiagen) according to the manufacturer's instructions. Quantitative reverse transcription polymerase chain reaction (PCR) was performed using iScript Reverse Transcriptase (Bio-Rad) and then a SsoFast EvaGreen Supermix (Bio-Rad) in a Mastercycler ep realplex apparatus (Eppendorf) with specific mouse oligonucleotides. *Cyp1a1* gene expression normalized to *Gapdh* was analyzed using primers previously described.^8^

### Histology and immunohistochemistry

Cross sections of the proximal jejunum were fixed in 10% formalin and embedded in paraffin. Small intestinal sections were stained with hematoxylin and eosin (H&E) and examined under light microscopy. We measured villus-to-crypt (V/C) ratios in a blinded fashion, as previously described.^8^^,^^20^ CD3-immunostained sections, performed as described previously with polyclonal rabbit anti-human CD3 (Dako),^8^^,^^20^^,^^23^were used to identify intraepithelial lymphocytes (IELs) in the epithelial layer. Counts were performed in 5 randomly selected villi by enumerating CD3^+^ IELs among enterocytes extending 10 cells in each direction from the villus tip, and results were expressed as CD3^+^ IELs per 100 enterocytes. Finally, a composite villus-to-crypt ratios and IEL (VCIEL) score was calculated according to the VCIEL scale as described previously.^24^

### *In vitro* batch cultures of small intestinal microbiota from NOD/DQ8 mice

*In vitro* cultures of small intestinal microbiota were performed in parallel with *in vivo* studies using samples collected from additional naïve NOD/DQ8 SPF mice (*n* = 8). Duodenal tissues (approximately 4 cm) from were homogenized in 800 uL of PBS in the Bullet Blender (Next Advance) at maximum speed for 5 min. Following homogenization, samples were centrifuged at 800 *g* for 1 min and the supernatant was collected. The process was repeated once, and the supernatants were pooled. 35 µL of the pooled supernatant or 35 µL of PBS was added to 1.8 mL of Brain Heart Infusion (BHI, Multicell) with 10 g/L of gelatin (Sigma) or casein (Sigma) as additional protein sources. Then, 1 × 10^6^ *S. boulardii* CFUs from rehydrated lyophilized preparations were immediately added to the batch culture. After 6 h of anaerobic culturing, bacterial suspensions were collected, and the samples were centrifuged at 4000 *g* for 5 min before supernatants and pellets were stored at −80 °C.

### CeD patients and duodenal aspirate collection

Patients attending the McMaster University Celiac Clinic were recruited after signing informed consent. Duodenal aspirates were collected from 5 patients (3F, 2M, median age: 46) with active CeD and 4 treated CeD patients (GFD > 2 y; 3F, 1M, median age: 43.5) (Supplementary Table S1). All patients with active CeD had a confirmed history of elevated serologic transglutaminase-2 (TG2) above 2 or 4 U/mL depending on the kit, and demonstrated histological changes with Marsh scores of 3a (*n* = 3), 3b (*n* = 1) and 1 (*n* = 1) (Supplementary Table S1). Patients on a GFD had a historical diagnosis of CeD, but normal TG2 and mild histological changes, with Marsh score 1 in all cases at inclusion (Supplementary Table S1). None of the participants were taking antibiotics or probiotics within a month of recruitment. Samples were obtained during an upper gastroduodenal endoscopy performed following overnight fasting. Intestinal mucus from the second portion of the duodenum was dislodged using a waterjet with sterile water, then luminal content was aspirated into a sterile mucus specimen trap (Medline). The study was registered at clinicaltrials.gov (ID: NCT06532110) and approved by the Hamilton Integrated Research Ethics Board (HiREB # 15311).

### *In vitro* batch cultures of duodenal microbiota from active and treated CeD patients

Microbiota from duodenal aspirates were grown anaerobically on BHI agar media plates supplemented with 0.5 g/L of L-cysteine, 10 mg/L of hemin and 1 mg/L of Vitamin K for 48 h. Plate pools were collected, and frozen glycerol stocks were prepared. Duodenal microbiota from the glycerol stocks were inoculated into liquid BHI and 35 uL of each sample was added to 1.8 mL of BHI with 10 g/L of casein. PBS, 1 × 10^6^ *S. boulardii* CFUs or 1 × 10^8^ *Lactobacillus reuteri* R12.22^25^ CFUs was immediately added to the batch culture. After 6 h of anaerobic culturing, bacterial suspensions were collected, and the samples were centrifuged at 4000 *g* for 5 min before supernatants and pellets were stored at −80 °C.

### Microbiota analysis

Total DNA was extracted from mouse proximal jejunums and culture-enriched plate pools, and data sequenced as previously described. The hyper-variable regions (V3–V4) of the bacterial 16S ribosomal RNA gene were amplified by polymerase chain reaction (PCR) and sequenced on the Illumina MiSeq platform by the McMaster Genomics facility. Sequences were then processed in R, version 4.4.2 using the package Divisive Amplicon Denoising Algorithm 2 (DADA2)^26^ and the SILVA reference database, version 138.1.^27^ FastTree 2^28^ was used to calculate a phylogenetic tree of sequences and data were explored using the phyloseq package.^29^ Beta-Diversity was calculated using the Aitchison distance^30^ and differences between groups were calculated using PERMANOVA. Taxonomic differences were evaluated using a generalized linear mixed model with a negative binomial distribution.^31^ Statistical differences between groups were identified using estimated marginal means^32^using the emmeans package. Functional predictions based on 16S rRNA sequences were completed using PICRUSt2^33^^,^^34^ annotated using the Kyoto Encyclopedia of Genes and Genomes (KEGG) database.^35^ Mouse 16S rRNA gene sequencing data was submitted to NCBI as PRJNA1309686 and PRJNA1439877. To verify the presence of *S. boulardii* in the feces of water-treated (*n* = 6 of 8) or *S. boulardii*-treated (*n* = 6 of 8) gluten-sensitized mice, we used qPCR of the DNA with the primers scTBP1O1 and scTBP1O1^36^ for *Saccharomyces* and 16S rRNA primers UnivF and UnivR^37^using the ΔΔCt method. Finally, we reanalyzed data from our previously published cohort consisting of healthy controls (*n* = 24) and active CeD (*n* = 22),^38^ and CeD on a GFD (*n* = 4),^39^ to investigate targeted relative abundance of *Lactobacillaceae* in small intestinal aspirates by 16S rRNA gene sequencing data. The data are publicly available; cohort procedures and original ethical approval are described in the primary reports.^38^^,^^39^

### Measurement of tryptophan levels

Free L-tryptophan levels in mouse duodenal samples and bacterial cultures were analyzed by the TR-FRET Bridge-IT L-Tryptophan Fluorescence Assay (Mediomics) according to the manufacturer's instructions.

### Measurement of AhR activity

The AhR activity in mouse duodenal contents, mouse bacterial cultures, and CeD microbiota cultures were measured using a luciferase reporter assay method, as described previously.^8^ Briefly, H1L1.1c2 cells, containing the dioxin response element-driven firefly luciferase reporter pGudLuc1.1, were seeded into a 96-well plate and stimulated with mouse duodenal suspensions or bacterial culture supernatants for 24 h. Luciferase activity was then measured using a luminometer.

### Measurement of total protein degradation

Total protein degradation was measured in mouse duodenal samples and bacterial samples using azocasein (Sigma-Aldrich) as described previously.^40^ Briefly, mouse duodenal suspensions and bacterial suspensions were incubated with 0.5% w/v azocasein substrate in 50 mM Tris-HCl buffer (pH 8.2) supplemented with 1 mM CaCl_2_, 50 mM NaCl, and 0.25% w/v Triton at 37 °C. The reaction was stopped with 10% trichloroacetic acid and absorbance was measured at 366 nm.

### Statistical analysis

GraphPad Prism 10 (GraphPad Software, USA) was used for preparation of graphs. Logarithmic data transformation was used when required and possible to achieve a normal distribution. For each *in vivo* response variable, using the R package lme4,^41^ we fitted a linear model for the experimental group in which sex was entered as an adjustment variable. Custom contrasts between the *S. boulardii* treatment and corresponding control were evaluated using estimated marginal means (emmeans)^32^ with Šidák adjustment for multiple-comparisons. Statistical analysis between paired *in vitro* samples was performed by paired *t*-test, and data with non-normal distribution were evaluated with Wilcoxon signed-rank test. Two-tailed statistical tests were used. Information regarding the exact analyses used are provided in each figure legend.

## Results

### *S. boulardii* decreases severity of gluten immunopathology in NOD/DQ8 mice

We first investigated the effects of *S. boulardii* in gluten-sensitized NOD/DQ8 mice (Figure 1a and Supplementary Figure S1a), a validated model of gluten sensitivity that develops moderate immunopathology upon sensitization with gluten and microbial adjuvant.^20^^,^^23^ We confirmed that *S. boulardii* was present in the feces of mice treated with the probiotic and was absent from the feces of water-treated mice (*p =* 0.03; Supplementary Figure S2). Gluten-sensitized mice exhibited lower V/C ratios compared with non-sensitized mice, and *S. boulardii* treatment improved V/C ratios by 23% in gluten-sensitized mice (*p* = 0.03; Figure 1b and Supplementary Figure S1c). *S. boulardii* also decreased CD3^+^ IEL counts by 40% in gluten-sensitized mice (*p* = 0.02; Figure 1c and Supplementary Figure S1d). Non-sensitized mice treated with *S. boulardii* or water had similar V/C ratios (Figure 1b and Supplementary Figure S1c) and IEL counts (Figure 1c and Supplementary Figure S1d). While no differences in VCIEL score were observed in non-sensitized mice treated with *S. boulardii* or water (Supplementary Figure S1b), gluten-sensitized mice treated with *S. boulardii* had an improved VCIEL score of 0.29 compared with water-treated gluten-sensitized mice (score: −1.63; *p* = 0.008; Supplementary Figure S1b). We did not observe any differences in gluten immunopathology between males and females treated with *S. boulardii* or water in non-sensitized or gluten-sensitized mice (Figure 1b and c and Supplementary Figure S1b). These findings indicate that *S. boulardii* ameliorates gluten-induced immunopathology in a mouse model of gluten sensitivity.

### *S. boulardii* improves gluten immunopathology involving the AhR pathway

To investigate the role of *S. boulardii* in AhR activation and its potential to further improve gluten sensitivity in our model, we measured the expression of key AhR genes in the small intestine of NOD/DQ8 mice. Intestinal expression of *Cyp1a1*, a downstream AhR target gene, was similar in non-sensitized mice treated with *S. boulardii* or water (Figure 1d). In gluten-sensitized mice, *Cyp1a1* expression was 86% higher with *S. boulardii* treatment than with water treatment, suggesting that the probiotic formulation activates the AhR pathway in the model (*p* = 0.05; Figure 1d).

To confirm whether *S. boulardii* improvement in gluten-immunopathology is mediated at least in part by AhR activation, we next blocked the pathway in gluten-sensitized mice using the pharmacological inhibitor CH-223191 (Figure 2a, Supplementary Figure S3a). While *S. boulardii*-treated mice increased V/C ratios by 27% versus water-treated mice (*p* < 0.0001), no improvement was observed in mice receiving the AhR inhibitor (Figure 2b and Supplementary Figure S3c). No differences in IEL counts were observed between groups (Figure 2c and Supplementary Figure S3d), but the composite VCIEL score was 7-fold higher in vehicle-treated mice receiving *S. boulardii* when compared to water treatment (*p* = 0.0001; Supplementary Figure S3b). No improvement in VCIEL score was observed in mice treated with the AhR inhibitor (Supplementary Figure S3b). In vehicle-treated mice, *S. boulardii* increased *Cyp1a1* expression by 82% versus no probiotic (*p* = 0.05; Figure 2d). This effect was absent in mice treated with the AhR inhibitor, where no differences in *Cyp1a1* expression were observed between *S. boulardii* and water-treated groups (Figure 2d). We did not observe differences in gluten immunopathology between males and females treated with *S. boulardii* or water in the vehicle-treated or AhR-inhibited mice (Figure 2b,c and Supplementary Figure S3b). These results suggest that *S. boulardii* improves gluten-induced immunopathology via the AhR pathway.

### *S. boulardii* increases small intestinal tryptophan levels and AhR activation

**Figure 3.**
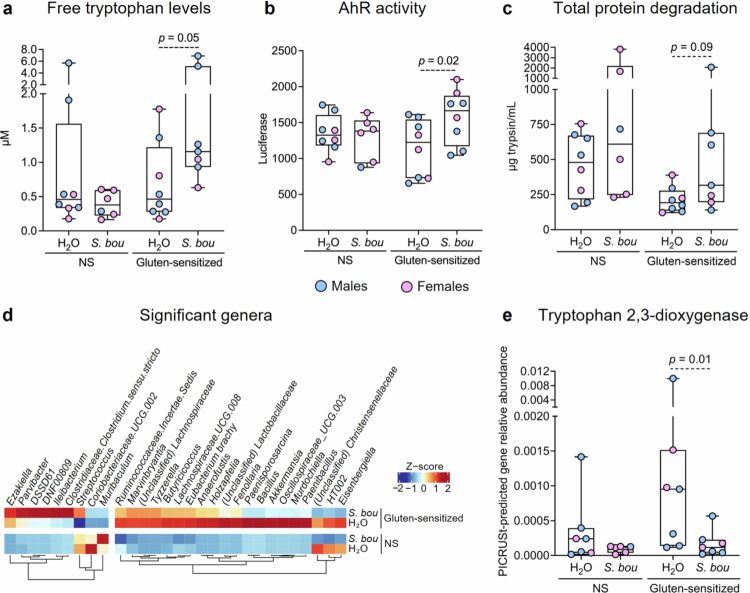
*S. boulardii* increases free tryptophan levels and AhR activation through effects on microbiota function and composition in gluten-sensitized NOD/DQ8 mice. (a) Free tryptophan levels, (b) AhR activity, and (c) total protein degradation in the small intestine of non-sensitized and gluten-sensitized mice treated with water or *S. bou*. (d) Heatmap representation of bacterial genera that were significantly affected by *S. bou* administration in gluten-sensitized mice, based on total relative abundance. (e) PICRUSt-predicted relative abundance of tryptophan 2,3-dioxygenase genes in non-sensitized and gluten-sensitized mice treated with water or *S. bou*. Data are presented as median with interquartile range and whiskers extending from minimum to maximum, where each dot represents an individual mouse (a–c, e). Significant differences were evaluated using a estimated marginal means and custom contrasts using emmeans.

Since AhR is activated by various tryptophan metabolites produced in the mucosa,^6^ we next quantified tryptophan levels in the small intestine and evaluated the ability of intestinal contents to activate AhR in our probiotic intervention model. In gluten-sensitized mice, free tryptophan levels were 2.5-fold higher in *S. boulardii* versus water-treated mice (*p* = 0.05; Figure 3a). In contrast, no differences in free tryptophan levels were observed in non-sensitized mice treated with *S. boulardii* or water (Figure 3a). Using a reporter cell line to measure AhR activation, we found a 37% increase in AhR activation by small intestinal contents of gluten-sensitized mice treated with *S. boulardii* versus water (*p* = 0.02; Figure 3b). Small intestinal contents from non-sensitized mice treated with *S. boulardii* or water had similar AhR activation capacity (Figure 3b).

Given that intestinal tryptophan levels can be influenced by protein degradation, and that *S. boulardii* has been shown to enhance overall protein degradation in the intestine,^18^ we then investigated total protein degradation in small intestinal contents. Gluten-sensitized mice treated with *S. boulardii* demonstrated a non-statistically significant trend (*p* = 0.09) for higher capacity to degrade protein versus those treated with water, which was not observed in non-sensitized mice (Figure 3c).

We next analyzed the small intestinal microbiota of non-sensitized and gluten-sensitized mice treated with *S. boulardii* or water. No significant differences in alpha-diversity were observed between groups. Beta-diversity demonstrated distinct clustering between non-sensitized and gluten-sensitized mice, with *S. boulardii* supplementation impacting profiles in non-sensitized mice (Supplementary Figure S4a). Administration of *S. boulardii* to gluten-sensitized mice increased the relative abundance of different taxa including *Clostridium*, a known AhR agonist producer^7^ (Figure 3d). PICRUSt-predicted analysis revealed that *S. boulardii* influenced microbial genes involved in tryptophan metabolism (Supplementary Figure S4b). Specifically, gluten-sensitized mice treated with *S. boulardii* showed a 91% reduction (*p* = 0.01; Figure 3e) in PICRUSt-predicted genes for tryptophan 2,3-dioxygenase, an enzyme that degrades indole rings, when compared to water-treated mice. Furthermore, AhR activation by small intestinal contents negatively correlated with PICRUSt-predicted genes for tryptophan 2,3-dioxygenase in gluten-sensitized mice (*R*^2^ = 0.22, *p* = 0.05; Supplementary Figure S4c), suggesting that AhR activation may be associated with predicted microbial indole availability. Altogether, these results suggest that *S. boulardii* supplementation leads to restoration of tryptophan metabolism by the microbiota and AhR activation in the small intestine.

### *S. boulardii* boosts mouse small intestinal microbial metabolic function

**Figure 4.**
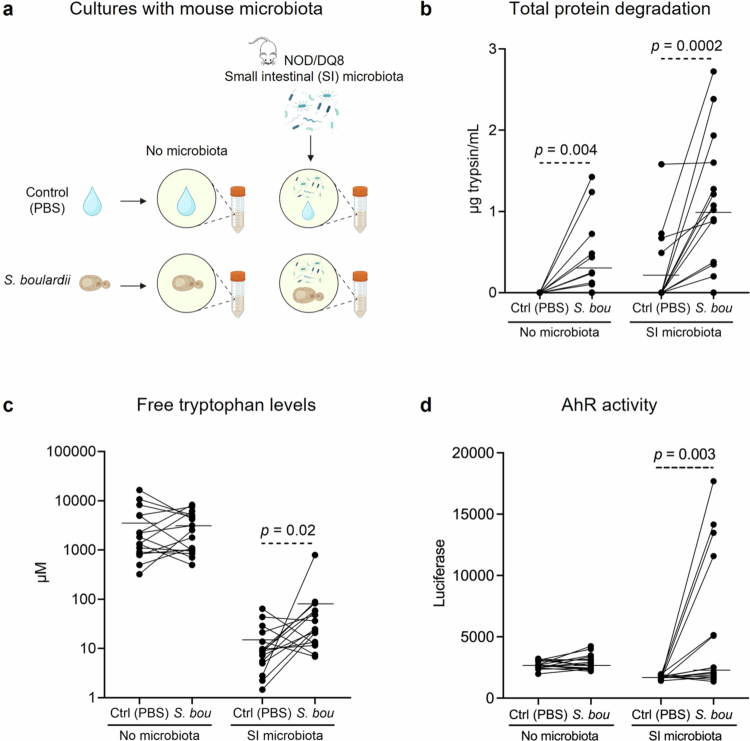
Microbiota is required for *S. boulardii* effects on protein digestion, free tryptophan production and AhR activation *in vitro*. (a) Experimental design for *in vitro* batch cultures with no microbiota or NOD/DQ8 mouse small intestinal microbiota with control (PBS) or *S. boulardii*, (b) Total protein degradation, (c) free tryptophan levels, and (d) AhR activity were measured in batch cultures. Data are presented as individual dots representing each mouse per culturing condition, where the lines indicate paired samples with the mean shown. Significant differences were evaluated using a two-tailed paired *t*-test or Wilcoxon signed-rank test.

To investigate *S. boulardii*-microbiota interactions on tryptophan metabolism, we conducted *in vitro* digestion experiments using small intestinal microbiota from non-sensitized SPF NOD/DQ8 mice, cultured with or without *S. boulardii,* in BHI media supplemented with protein (Figure 4a). *S. boulardii* increased total protein degradation from 0 µg to 0.31 µg trypsin/mL of culture supernatant (*p* = 0.004; Figure 4b). This effect was further amplified, showing a 3.5-fold increase when mouse microbiota was present in the culture (*p* = 0.0002; Figure 4b). Free tryptophan levels increased 4.5-fold when *S. boulardii* was added to the culture in the presence of microbiota (*p* = 0.02), and no differences in tryptophan levels were observed in cultures without microbiota (Figure 4c). This was paralleled by a two-fold increase in AhR activity in cultures combining *S. boulardii* with mouse microbiota, compared to microbiota alone without probiotic (*p* = 0.003; Figure 4d). No differences in AhR activity were observed in the absence of microbiota (Figure 4d). Analysis of the mouse microbiota composition used for the *in vitro* cultures reveal that there is a high relative abundance of *Lactobacillaceae* (Supplementary Figure S5a left), a bacterial family known to produce AhR agonists.^8^ These findings suggest that interactions between *S. boulardii* and the small intestinal microbiota regulate tryptophan availability and promote AhR activation.

### *S. boulardii* and *L. reuteri* increase AhR activity of CeD microbiota

**Figure 5.**
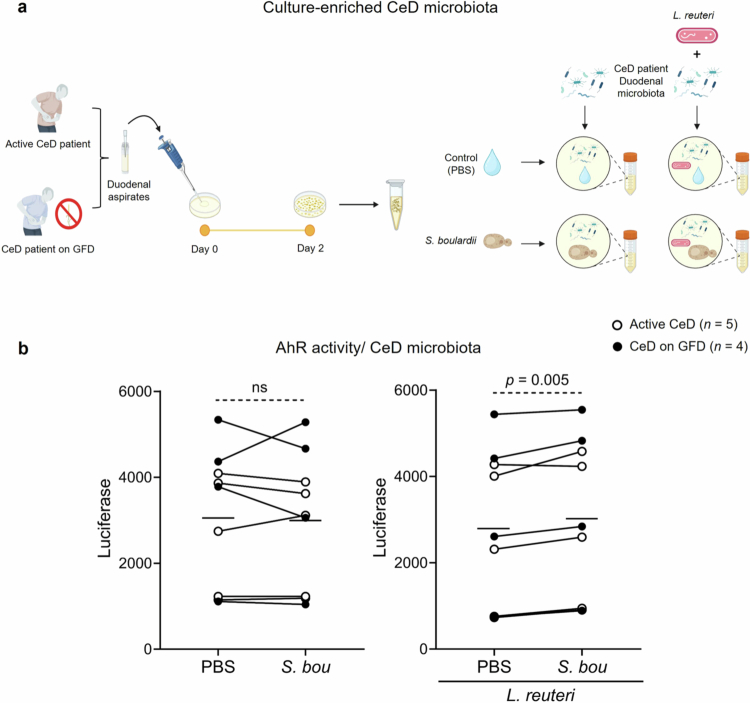
Combination of *S. boulardii* and *L. reuteri* increase AhR activity in duodenal CeD microbiota co-culture *in vitro*. (a) Experimental design for *in vitro* batch cultures with 48 h culture-enriched microbiota from duodenal aspirates of active CeD patients and patients on a GFD, with the addition of *L. reuteri* in media with control (PBS) or *S. boulardii*. (b) AhR activity of batch cultures with CeD microbiota. Open dots represent cultures containing microbiota from active CeD patients and closed dots represent cultures containing microbiota from CeD patients on a GFD. Data are presented as a representative of 3 independent experiments, where the lines indicate paired samples with the mean shown. Significant differences were evaluated using a two-tailed paired *t*-test.

To investigate whether *S. boulardii* also enhances AhR activation in the presence of human CeD small intestinal microbiota, we obtained duodenal aspirates from patients with active or treated CeD (Supplementary Table S1). Because the duodenum is a low microbial biomass site, we performed a culture-enriched step before co-incubation with *S. boulardii* (Figure 5a). In contrast with cultures using naïve mouse small intestinal microbiota, S. *boulardii* did not increase AhR activity when cultured with CeD microbiota (Figure 5b). Because patients with CeD were previously shown to have impaired microbial metabolism of tryptophan and AhR activation in the duodenum,^8^ we hypothesized that unlike naïve mouse samples, patient samples may be depleted of indole producers. We thus reanalyzed publicly available data from our previous studies,^38^^,^^39^ with a focus on *Lactobacillaceae*. Patients with active and treated CeD had a lower relative abundance of *Lactobacillaceae* compared with healthy controls (Supplementary Figure S5a right). More than 40% of patients with active CeD (*n* = 22) and 20% of patients with CeD on a GFD (*n* = 4) lacked *Lactobacillaceae* in their small intestinal aspirates (Supplementary Figure S5b). We then designed experiments to test the effect of *S. boulardii* and *Lactobacillus reuteri* (*L. reuteri*), a species known to produce AhR agonists,^8^ on the AhR activity of CeD microbiota *in vitro* (Figure 5a). *S. boulardii* increased AhR activation of CeD microbiota enriched with *L. reuteri* (*p* = 0.005 vs without *S. boulardii*; Figure 5b). *L. reuteri* did not increase AhR activation in the absence of microbiota, with or without *S. boulardii* (Supplementary Figure S6). These results suggest that *S. boulardii* can potentiate AhR activation in the presence of microbiota and especially when indole-producing bacteria, reported to be depleted in CeD, are present.

## Discussion

The AhR pathway regulates intestinal homeostasis through immune and barrier function,^7^ and is activated by microbial metabolites of tryptophan.^6^ Impairment of this pathway has been demonstrated in clinical conditions such as inflammatory bowel disease,^42^ irritable bowel syndrome (IBS)^21^ and CeD.^8^ In CeD, the GFD only partially restores the AhR pathway,^8^ suggesting that it may be a viable target for therapeutic modulation. Here we provide preclinical evidence of AhR pathway enhancement by the probiotic *S. boulardii* in genetically predisposed mice sensitized with gluten. We demonstrate through pharmacological inhibition of AhR that the beneficial effect of *S. boulardii* is mediated in part through this pathway. Finally, we identify a novel *S. boulardii*-microbiota interaction, by which the yeast probiotic boosts the tryptophan metabolic capacity of the small intestinal microbiota of mice and patients with CeD, although the latter requires enrichment with tryptophan metabolizers, often depleted in patients.^43-46^ The results provide preclinical evidence of gluten-immunopathology improvement by the probiotic yeast *S. boulardii* through indirect modulation of bacterial tryptophan metabolism, known to be impaired in some patients with CeD.

Using a validated mouse model of gluten sensitivity,^20^^,^^23^ we found that administering *S. boulardii* to gluten-sensitized mice increased free tryptophan levels and enhanced AhR activation in the small intestinal contents. These changes were associated with improvement of V/C ratios and decreased IEL counts compared to mice that did not receive *S*. *boulardii* treatment. Pharmacological inhibition of AhR confirmed that the beneficial effect of *S. boulardii* involves this pathway, which is consistent with a previous study in a mouse model of IBS.^21^

Microbial tryptophan metabolites (indoles) are the primary agonists of the AhR and are generated through the metabolism of tryptophan, such as that resulting from the breakdown of dietary proteins.^6^ We found an increase in protein degradation in gluten-sensitized mice treated with *S. boulardii*. This is in line with previous work demonstrating that *S. boulardii* may influence the production of host enzymes through trophic factors, and secrete its own enzymes,^18^ resulting in increased availability of tryptophan to serve as a precursor for indole-producing microbes. Additionally, upon analysis of microbial predicted function, we found that *S. boulardii* reduced the number of predicted microbial tryptophan 2,3-dioxygenase gene counts in gluten-sensitized mice. This enzyme degrades the indole ring of tryptophan, which may decrease the pool of metabolites available for indole biosynthesis and activation of the AhR pathway. This is supported by a recent study showing that *in vitro, S. boulardii* enhances levels of indole-3-propionic acid, an AhR agonist produced by the microbiota.^15^ Unfortunately, individual indoles were below the detection limits of metabolomic quantification in our study, which may reflect technical limitations associated with the small intestinal environment. However, activation of the AhR pathway is supported by increased *Cyp1a1* expression, a downstream target of AhR, as well as by functional activation of an AhR reporter cell line in response to small intestinal contents. In addition, we found increased relative abundance of different bacteria including *Clostridium*, a well-known taxon implicated in indole production.^7^ Our study reveals two potential mechanisms mediated by interactions between *S. boulardii* and the microbiome, through enhanced protein degradation and/or reduced microbial enzymatic degradation, which results in increased tryptophan availability as a substrate for indole production and AhR activation.

To investigate *S. boulardii*-microbiota interactions in the production of AhR ligands, we used an *in vitro* approach. Similar to *in vivo* results, *S. boulardii* enhanced protein degradation, and this was observed both with and without the presence of small intestinal microbiota from naïve NOD/DQ8 mice, indicating the function originates from the probiotic yeast. Despite this, *S. boulardii* increased free tryptophan levels and AhR activity only in the presence of small intestinal microbiota. This suggests that in the absence of microbiota, *S. boulardii* may metabolize tryptophan for its own function, leading to a reduction in AhR ligands and no AhR activation. On the other hand, the enzymatic activity of small intestinal microbiota is required to complete the digestion of protein substrates, resulting in increased free tryptophan availability. Our results reveal that the enhanced AhR activation mediated by *S. boulardii* is thus dependent on the presence of small intestinal microbiota and its underlying metabolic function, highlighting a novel beneficial *S. boulardii*-microbiota interaction.

To investigate whether the AhR activation capacity of microbiota from patients with CeD is also enhanced by *S. boulardii*, we cultured patient microbiota from duodenal aspirates *in vitro* alone or in co-culture with *S. boulardii.* An increase in AhR activity was only observed when the CeD microbiota-*S. boulardii* cultures were supplemented with *L. reuteri*. This is in line with previous reports that CeD microbiota is depleted of indole metabolizers such as *Lactobacillus.*^43-46^ Moreover, in the absence of CeD microbiota, co-culture of *L. reuteri* with *S. boulardii* did not increase AhR activity, emphasizing the complexity of interactions between endogenous microbiota and the probiotic. Our results suggest that the metabolic function of the CeD small intestinal microbiota can be boosted with probiotic combinations such as *S. boulardii* and *L. reuteri* to increase AhR activation, promoting restoration of intestinal homeostasis.

Our study has several limitations. AhR inhibitor treatment was necessarily shortened because the loss of AhR expression causes impaired intestinal barrier function in mice,^22^ which may have limited worsening immunopathology; however, the key mechanistic finding-that inhibition abolished the probiotic benefit-remained consistent. Together with our prior work showing that dietary tryptophan rescues AhR agonist production in gluten-sensitized mice,^8^ the present data suggest that naïve and gluten-sensitized mice differ in tryptophan microbial metabolic capacity, which may affect AhR restoration response to tryptophan supplementation^8^ or probiotics, as in the current study. In addition, the *in vitro* batch culture system does not fully capture the *in vivo* intestinal environment, and CeD microbiota experiments were limited by small sample size. However, the use of small intestinal rather than fecal microbiota^38^ increases translatability, and the results should encourage intervention in clinical trials.

In conclusion, our results show that *S. boulardii* reduces gluten immunopathology in gluten-sensitized NOD/DQ8 mice in part through activation of the AhR pathway. These beneficial effects require the interaction between *S. boulardii* and the microbiota to increase free tryptophan and AhR activation, as demonstrated both *in vivo* and *in vitro*. We show that AhR activation capacity of CeD patient microbiota can be increased with *S. boulardii*, although enrichment of indole producers such as *L. reuteri* is required to achieve a significant difference. Currently, a strict, life-long GFD is the only treatment available to patients with CeD, however many patients still experience persistent symptoms and intestinal inflammation.^47^ While probiotics have shown some symptomatic benefit in CeD patients, the evidence is low, and specific mechanisms relevant to CeD have not been studied.^12^ Here, we identify a specific mechanistic pathway mediated by *S. boulardii* CNCM I-745, a long-time used probiotic strain with demonstrated safety, which should encourage clinical testing to prevent CeD or as an adjuvant therapy to the GFD in non-responsive patients.

## Supplementary Material

Supplementary material Supplementary Figure S3.jpg

Supplementary material Supplementary Figure S2.jpg

Supplementary material Supplementary Figure S1.jpg

Supplementary material Supplementary Figure S6.jpg

Supplementary material Supplementary Figure S5.jpg

Supplementary material Supplementary Table S1.jpg

Supplementary Figure S4.jpg Supplementary Figure S4.jpg

Supplementary material Supplementary Figure 1

## Data Availability

All sequencing data have been deposited in the Sequence Read Archive. 16S rRNA gene sequencing data used in this study can be accessed under BioprojectID 1309686, BioProject ID PRJNA812940, Bioproject ID PRJNA1256891 and PRJNA1439877 at http://www.ncbi.nlm.nih.gov/bioproject/1309686, http://www.ncbi.nlm.nih.gov/bioproject/812940, http://www.ncbi.nlm.nih.gov/bioproject/1256891 and http://www.ncbi.nlm.nih.gov/bioproject/1439877.
