# Supplementary figures and images for: Saccharomyces boulardii CNCM I-745 synergizes with the small intestinal microbiota to boost AhR signaling in celiac disease
Source: Gut Microbes. 2026 May 1;18(1):2664640. doi: 10.1080/19490976.2026.2664640 (PMC13138080; doi:10.1080/19490976.2026.2664640)

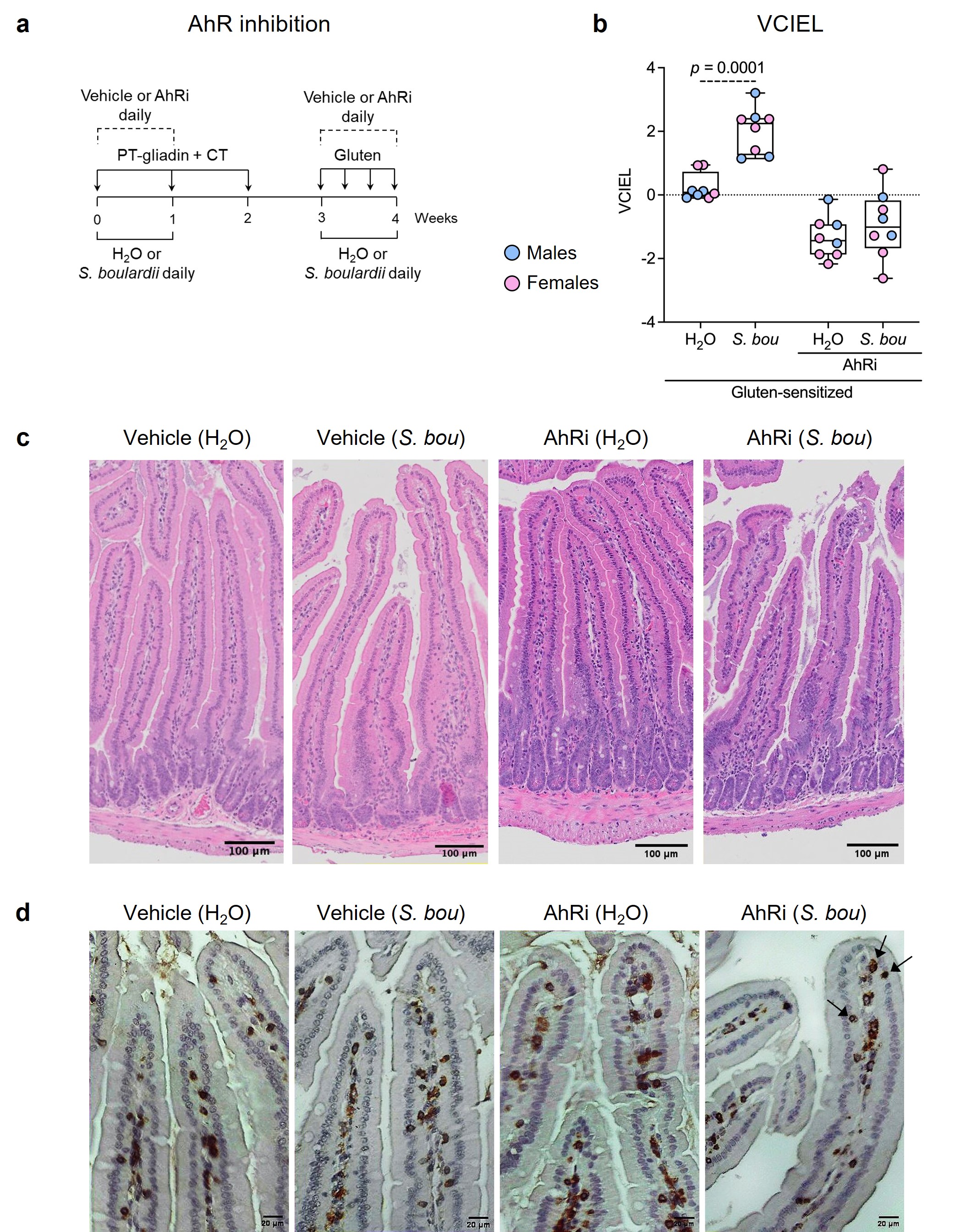

Supplement: Supplementary material — Supplementary Figure S3.jpg [file KGMI_A_2664640_SM7165.jpg]

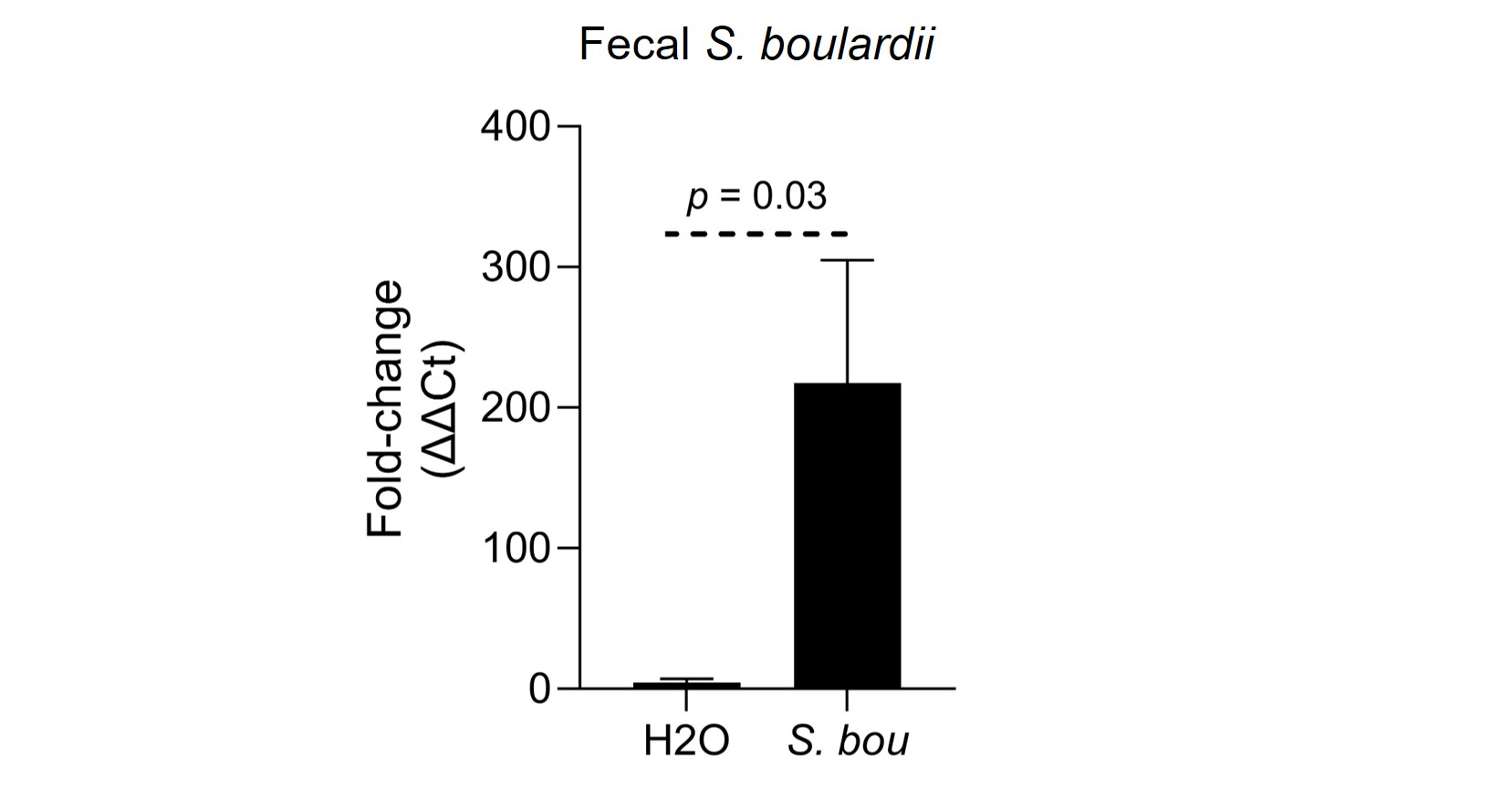

Supplement: Supplementary material — Supplementary Figure S2.jpg [file KGMI_A_2664640_SM7166.jpg]

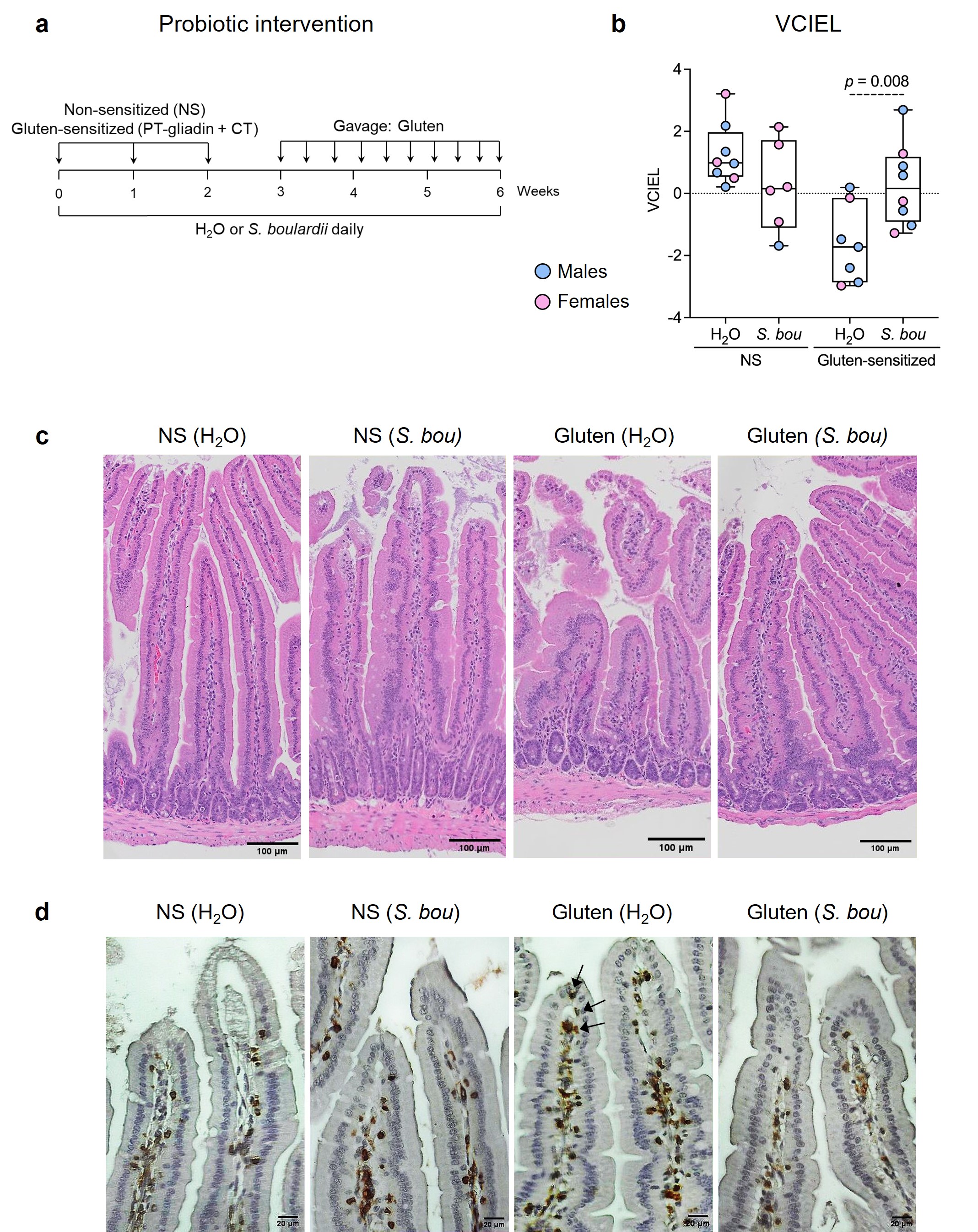

Supplement: Supplementary material — Supplementary Figure S1.jpg [file KGMI_A_2664640_SM7167.jpg]

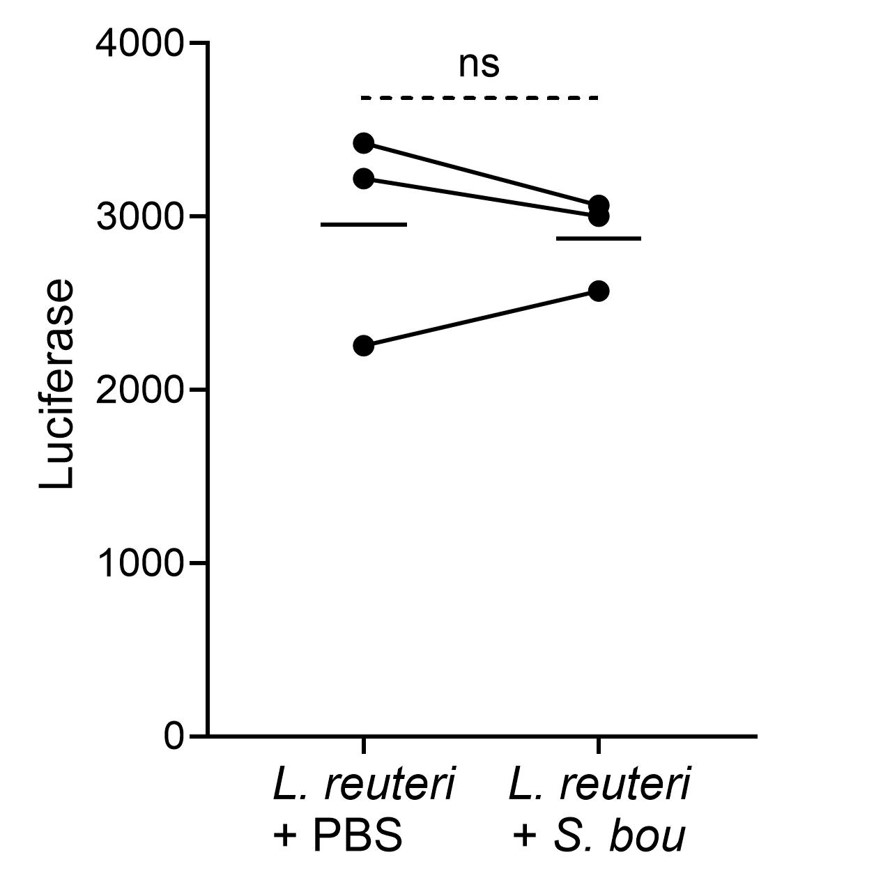

Supplement: Supplementary material — Supplementary Figure S6.jpg [file KGMI_A_2664640_SM7168.jpg]

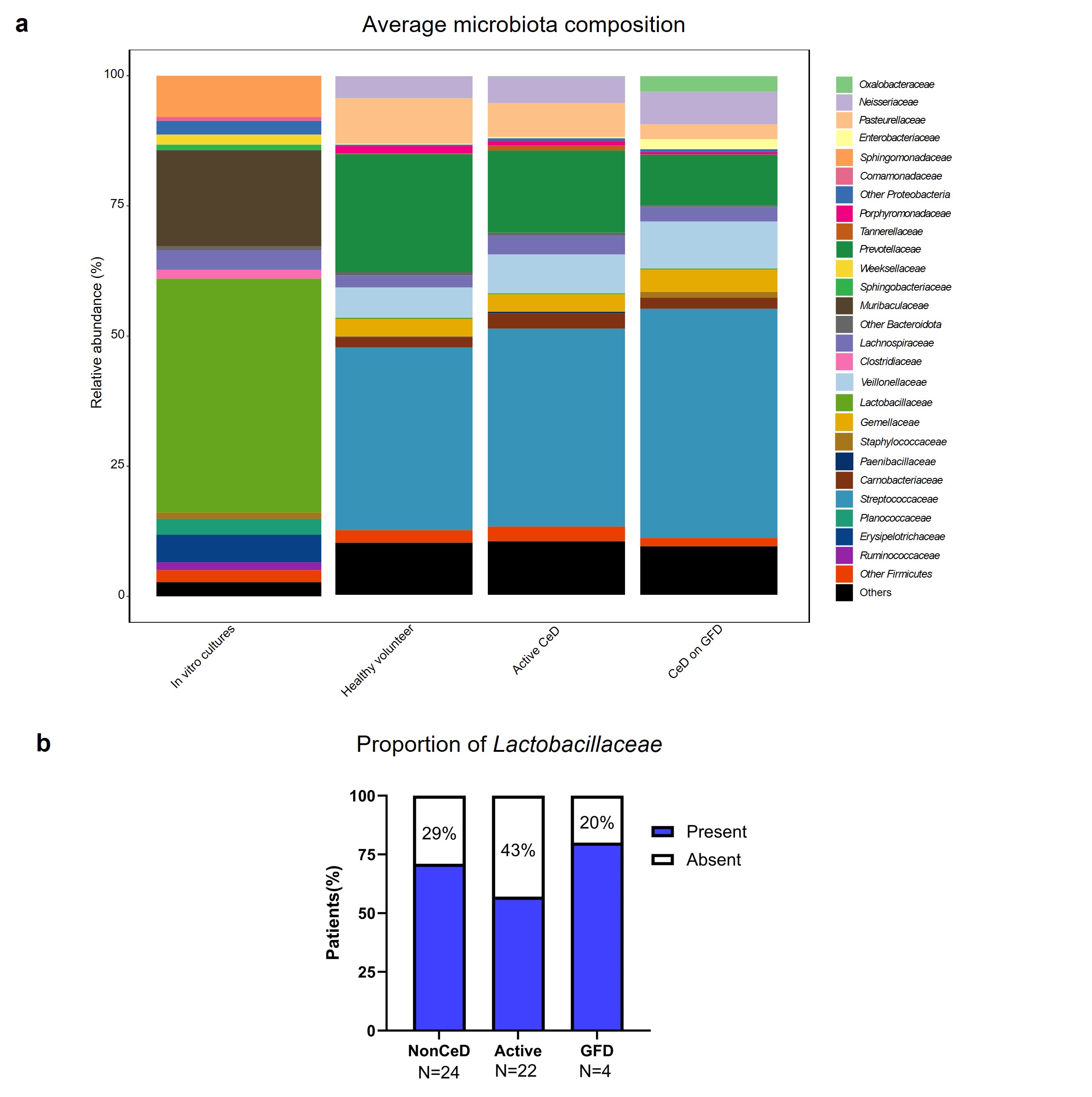

Supplement: Supplementary material — Supplementary Figure S5.jpg [file KGMI_A_2664640_SM7169.jpg]

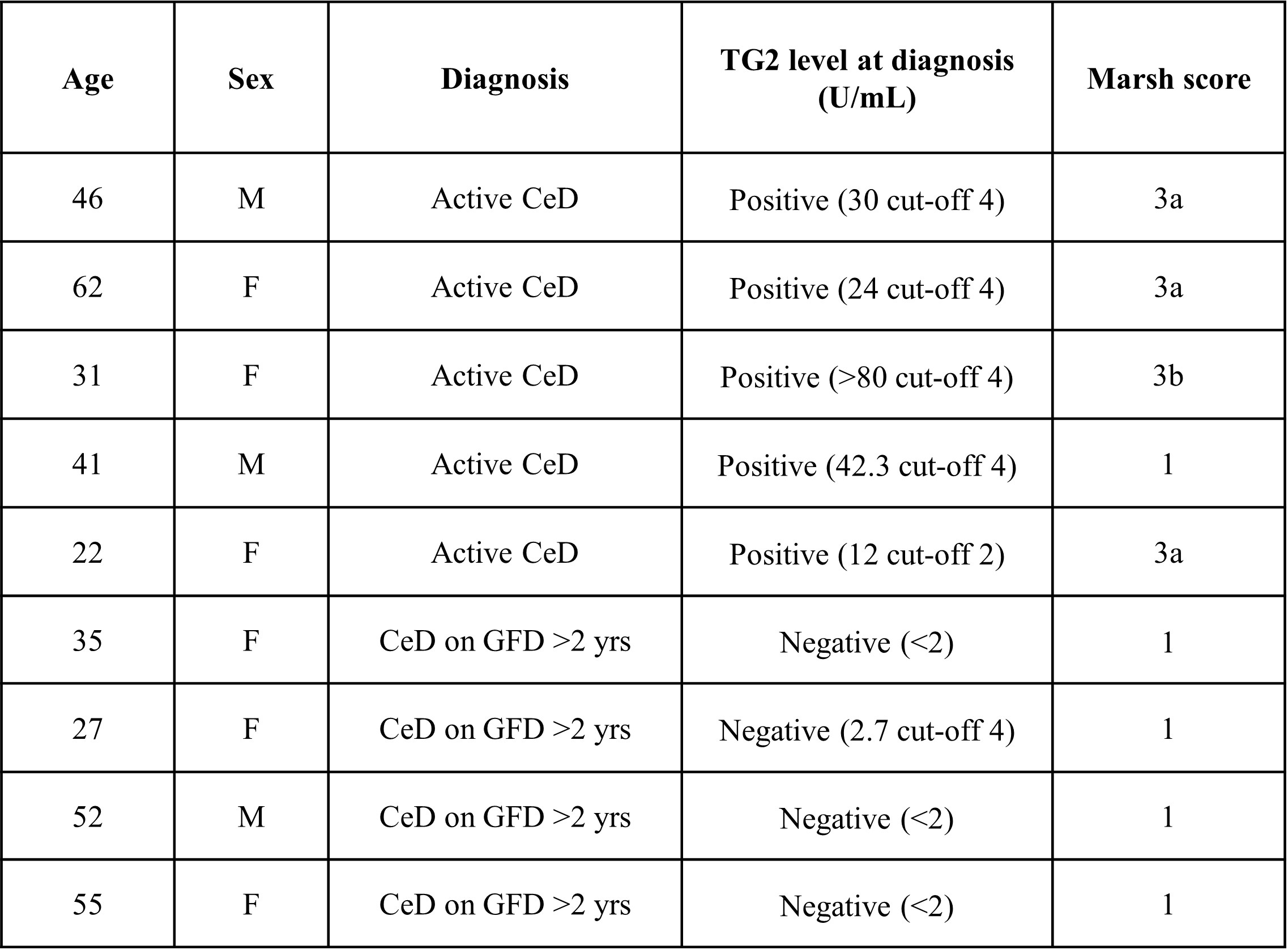

Supplement: Supplementary material — Supplementary Table S1.jpg [file KGMI_A_2664640_SM7170.jpg]

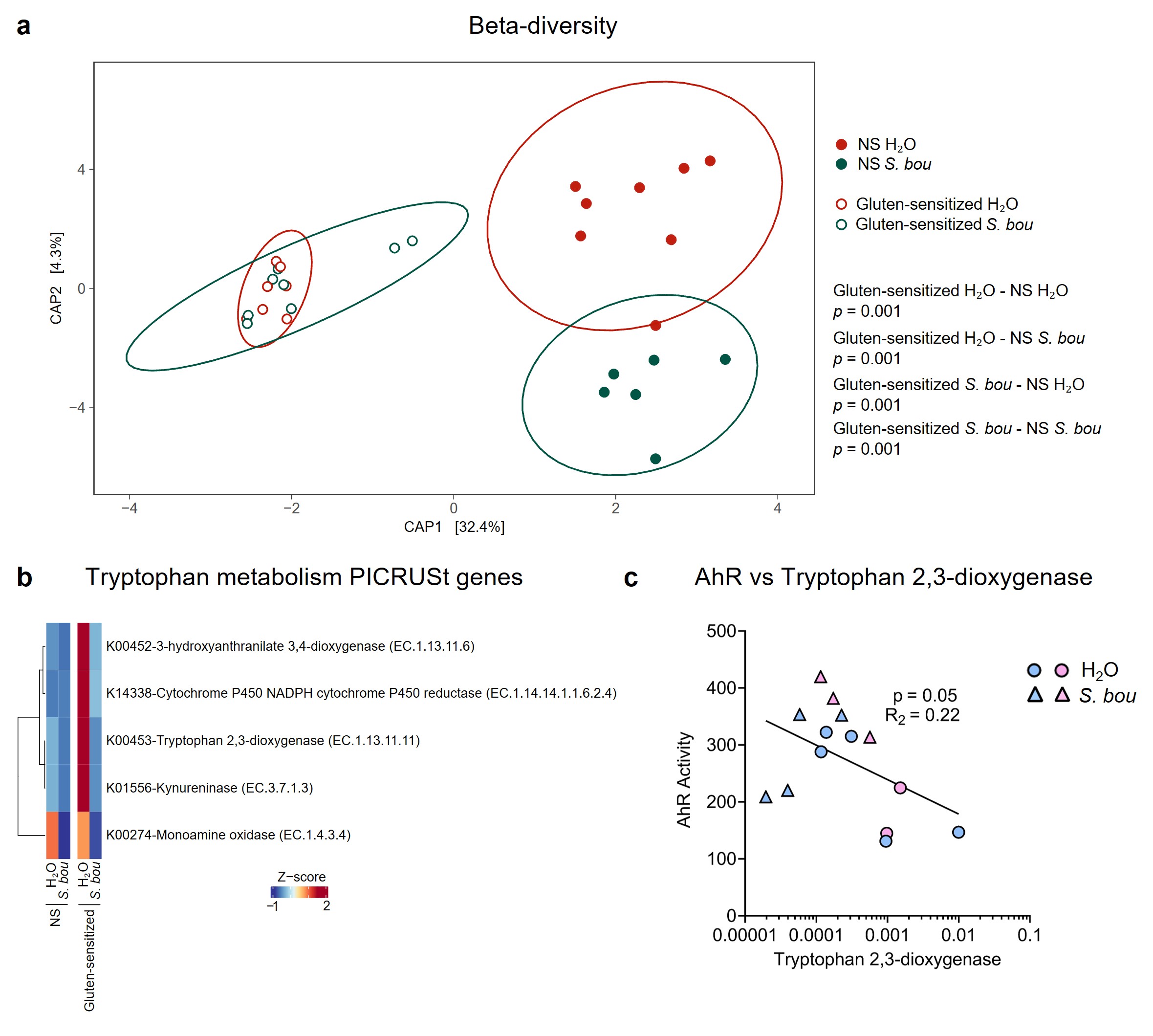

Supplement: Supplementary Figure S4.jpg [file KGMI_A_2664640_SM7171.jpg]
