# Supplementary material for: Saccharomyces boulardii CNCM I-745 synergizes with the small intestinal microbiota to boost AhR signaling in celiac disease
Source: Gut Microbes. 2026 May 1;18(1):2664640. doi: 10.1080/19490976.2026.2664640 (PMC13138080; doi:10.1080/19490976.2026.2664640)
Supplement: Supplementary material — Supplementary Figure 1 [file KGMI_A_2664640_SM1518.docx]

**Supplementary Figure 1**. Histopathology of non-sensitized and gluten-sensitized NOD/DQ8 mice treated with *S. boulardii*. (a) Experimental design for *S. boulardii* treatment of NOD/DQ8 mice. NOD/DQ8 mice were not sensitized (non-sensitized) or sensitized with PT-gliadin and cholera toxin once a week for 2 weeks, then 1 week following sensitization mice were gavaged with gluten every 2 days for 3 weeks (gluten-sensitized). Non-sensitized or gluten-sensitized mice were gavaged with water (H_2_O) or *Saccharomyces boulardii* (*S. bou*) daily for 6 weeks. (b) VCIEL scale for non-sensitized or gluten-sensitized mice treated with water or *S. bou*. Data are presented as median with interquartile range and whiskers extending from minimum to maximum where each dot represents an individual mouse. Significant differences were evaluated using estimated marginal means and custom contrasts using emmeans. (c) Representative hematoxylin and eosin (H&E)-stained small intestinal sections. *Scale bar*, 100 µm. (d) Representative CD3^+^ stained sections of the small intestine, where CD3^+^ IELs are stained in brown. Scale bar, 20 µm.

**Supplementary Figure 2.** Quantification of *S. boulardii* in feces of gluten-sensitized NOD/DQ8 mice treated with H_2_O or probiotic. Significant differences were evaluated using two-tailed unpaired *t* test.

**Supplementary Figure 3**. Histopathology of gluten-sensitized NOD/DQ8 mice treated with AhR inhibitor CH-223191 and *S. boulardii*. (a) Experimental design for AhR inhibition of NOD/DQ8 mice. Gluten-sensitized NOD/DQ8 mice were gavaged daily for 1 week of the sensitization phase and challenge phase with *S. boulardii* (*S. bou*) or water (H_2_O), and with vehicle or CH-223191 (AhRi). (b) VCIEL scale for mice treated with vehicle or AhRi receiving water or *S. bou*. Data are presented as median with interquartile range and whiskers extending from minimum to maximum where each dot represents an individual mouse. Significant differences were evaluated using estimated marginal means and custom contrasts using emmeans. (c) Representative hematoxylin and eosin (H&E)-stained small intestinal sections. Scale bar, 100 µm. (d) Representative CD3^+^ stained sections of the small intestine, where CD3^+^ IELs are stained in brown. Scale bar, 20 µm.

**Supplementary Figure 4**. *S. boulardii* influences predicted microbial tryptophan metabolism and microbiota composition. (a) Beta-diversity of small intestinal microbiota profiles of non-sensitized and gluten-sensitized mice treated with water or *S. bou* using Bray-Curtis distances. (b) Differential abundance testing was determined to compare PICRUSt-predicted tryptophan metabolism genes associated with *S. bou* administration in non-sensitized and gluten-sensitized NOD/DQ8 mice. (c) Correlation between duodenal AhR activity and PICRUSt-predicted genes for tryptophan 2,3-dioxygenase in gluten-sensitized mice treated with water or *S. bou*. Each dot represents an individual mouse where circles represent mice treated with water and triangles represent mice treated with *S. bou*.

**Supplementary Figure 5.** Microbiota composition of naïve NOD/DQ8 mice used for *in vitro* cultures and replotting of previous cohorts^38, 39^. (a) Left - Average relative abundance at the family level of *in vitro* cultures with naïve NOD/DQ8 microbiota. Right – Average relative abundance at the family level of healthy volunteer, patients with active CeD, and CeD patients on GFD duodenal aspirate microbiota. (b) Proportion of *Lactobacillaceae* in healthy volunteer (NonCeD), patients with active CeD, and CeD patients on GFD duodenal aspirate microbiota.

**Supplementary Figure 6**. Combination of *S. boulardii* and *L. reuteri* does not increase AhR activity in co-cultures without duodenal CeD microbiota. AhR activity of batch cultures with *L. reuteri* and control (PBS) or *S. boulardii* was measured. Data are presented as individual dots per replicate of 1 representative experiment where the lines indicate paired samples with the mean shown.

**Supplementary Table S1. Individual patient demographic information.**
